# Bacteriophages Promote Metabolic Changes in Bacteria Biofilm

**DOI:** 10.3390/microorganisms8040480

**Published:** 2020-03-28

**Authors:** Marina Papaianni, Paola Cuomo, Andrea Fulgione, Donatella Albanese, Monica Gallo, Debora Paris, Andrea Motta, Domenico Iannelli, Rosanna Capparelli

**Affiliations:** 1 Department of Agricultural Sciences, University of Naples Federico II, 80055 Portici, Naples, Italy; marina.papaianni@unina.it (M.P.); paola.cuomo@unina.it (P.C.); andrea.fulgione@unina.it (A.F.); iannelli@unina.it (D.I.); 2 Istituto Zooprofilattico Sperimentale del Mezzogiorno (IZSM), 80055 Portici, Naples, Italy; 3 Department of Industrial Engineering University of Salerno, 84084 Fisciano, Italy; dalbanese@unisa.it; 4 Department of Molecular Medicine and Medical Biotechnology, University of Naples Federico II, 80131 Naples, Italy; monica.gallo@unina.it; 5 Institute of Biomolecular Chemistry, National Research Council, 80078 Pozzuoli, Naples, Italy; dparis@icb.cnr.it

**Keywords:** bacteriophages, biofilm, NMR, metabolomics

## Abstract

Bacterial biofilm provides bacteria with resistance and protection against conventional antimicrobial agents and the host immune system. Bacteriophages are known to move across the biofilm to make it permeable to antimicrobials. Mineral hydroxyapatite (HA) can improve the lytic activity of bacteriophages, and, together with eicosanoic acid (C20:0), can destroy the biofilm structure. Here, we demonstrate the efficacy of the combined use of phage, HA and C20:0 against *Xanthomonas* *campestris* pv *campestris* (*Xcc*) biofilm. We used nuclear magnetic resonance (NMR)-based metabolomics to investigate the molecular determinants related to the lytic action, aiming at identifying the metabolic pathways dysregulated by phage treatment. Furthermore, we identified specific markers (amino acids, lactate and galactomannan) which are involved in the control of biofilm stability. Our data show that Xccφ1, alone or in combination with HA and C20:0, interferes with the metabolic pathways involved in biofilm formation. The approach described here might be extended to other biofilm-producing bacteria.

## 1. Introduction

The concern about bacterial resistance to antibiotics and microbial biofilm production is rapidly increasing. The latest data collected by the European Centre for Disease Prevention and Control highlight a persistent increase of antibiotic-resistant bacteria in the clinical area, as well as in the food industry and agriculture [1].

Biofilm production is the response of bacteria to adverse environmental conditions [2], such as the presence of antibiotics, or the need to establish a chronic colonization [3,4,5]. The creation of a (thick) biofilm represents a physical barrier to antibiotics, and structural modifications can also develop in membrane composition and in the antibiotics’ targets [6,7]. At present, microbial biofilm production represents a major economic and clinical problem, and its prevention and treatment are therefore a major concern.

Bacteriophages (phages) are viruses infecting bacteria, and in contrast to many antibiotics [8], phages can selectively lyse bacteria protected by the biofilm [9,10,11]. In addition, phages are species-specific, and therefore can be used to target pathogenic bacteria without disturbing non-harmful commensal bacteria [12]. At present, the use of phages to control pathogens in the food industry and agriculture is rather limited [13]. Moreover, since antibiotics have lost much of their power against bacteria, phage therapy may acquire a major role in combating resistant bacterial strains. Consequently, understanding the molecular determinants of phage–host interactions appears to be an essential step for a safe application of the therapy [14]. Recently, metabolomics analysis has suggested that the molecular response to phage infection is specific, as the molecular interactions taking place depend upon the phage and host bacterial strain [15,16]. Metabolomics is the systematic identification and quantification of all metabolites (i.e., the metabolome) in a biological matrix. Metabolomics is particularly effective to investigate how phages act against bacteria during infection [14,15]. Currently, nuclear magnetic resonance (NMR)-based metabolomics is widely used to define alteration of metabolic profiles, unambiguously recognizing biomarkers that characterize different systems biology states.

In this paper, by using NMR-based metabolomics, we investigated the molecular determinants related to the action of the phage against the *Xanthomonas campestris* pv. *campestris* (*Xcc*) biofilm. *Xcc* is a Gram-negative bacterium distinguished into several pathovars with specific host range. Xcc is the causal agent of crucifer (including broccoli, cabbage, cauliflower, radish, etc.) black rot disease, causing yield loss in agricultural production world-wide [17]. As with many phytopathogenic bacteria, Xcc produces a range of factors that help the bacterium to parasitize the host [18]. The exopolysaccharides can obstruct the xylem vessels, causing tissue necrosis and leaf wilting [19]. In particular, we aimed at characterizing the metabolic pathways dysregulated by phage treatment, which could become the possible targets, as well as providing an indication of the efficacy of the treatment. Previous studies have demonstrated that hydroxyapatite (HA) enhances the activity of phages. The low degree of crystallinity and the presence of carbonate ions in the crystal structure make HA extremely reactive in biological systems and particularly suitable to interact and transport bacteriophages [20]. The eicosanoic acid (C20:0) weakens the bacterial biofilm structure [21,22,23], and phage Xccφ1 can control *Xcc* infection in plants (submitted by Papaianni). Here, we demonstrated that the simultaneous use of HA, C20:0 and Xccφ1 destroys the *Xcc* biofilm structure, identifying specific biomarkers involved in the control of biofilm stability.

## 2. Material and Methods

### 2.1. Isolation and Growth of Xcc Phages

Ten grams of rhizospheric soil from *Brassica oleracea* plants with black rot symptoms (characteristic of *Xcc* infection) were suspended in 15 mL of nutrient broth (Sigma Aldrich, Milan, Italy) and shacked for 30 min at 24 °C. Soil sediment was removed by centrifugation (5000 rpm for 10 min), and individual supernatants (15 mL) were transferred into sterile flasks. Forty milliliters of 10^6^ colony-forming units (CFU) per ml of *Xcc* bacteria in exponential growth phase were added to each flask. Flasks were incubated overnight at 24 °C. Cultures were treated with chloroform, clarified by centrifugation, and filtered through Millipore 0.22 µm-pore-size membrane filters (MF-Millipore, Darmstadt, Germany). Filtrates were tested for the presence of *Xcc*-specific phages as described [20].

### 2.2. Eicosanoic Acid Activity against Biofilm

The eicosanoic acid (C20:0) activity was tested by the crystal violet staining test [24]. Individual wells of a polystyrene 96 flat-well plate (Falcon) were spotted with 200 µL of *Xcc* bacteria (10^6^ colony-forming units per mL). To facilitate bacterial attachment, the plates were incubated for 72 h at 24 °C without shaking. C20:0 was then added (60 µg/mL, 120 µg/mL, or 240 µg/mL per well), and again incubated for 8 h. After treatment, planktonic cells were gently removed, and the wells washed three times with water. For NMR studies, C20:0 was used at the lowest concentration.

### 2.3. Preparation of Supernatants for Metabolic Analysis

To facilitate biofilm formation, the *Xcc* bacterial suspension was distributed in Erlenmeyer flasks (50 mL/flask) and incubated for 72 h at 24 °C under a static condition. Next, 5 mL of phages (10^8^ plaque forming-units (PFU)/mL), acid (30 µg/mL) or Xccφ1+HA+C20:0 (10^8^ PFU/mL, 5 mg/mL and 30 µg/mL respectively) were added to each flask. After 3 h incubation at 24 °C, the cultures were collected, centrifuged (13,000 rpm for 20 min) and the supernatants stored at +4 °C for NMR analysis.

### 2.4. NMR Spectroscopy

NMR spectra were recorded on a Bruker Avance III-600 MHz spectrometer (Bruker BioSpin GmbH, Rheinstetten, Germany), equipped with a TCI CryoProbe^TM^ fitted with a gradient along the Z-axis, at a probe temperature of 27 °C. One-dimensional (1D) proton spectra were acquired at 600 MHz by using the excitation sculpting sequence [25]. Two-dimensional (2D) total correlation spectroscopy (TOCSY) spectra [26,27] were acquired using the MLEV-17 a broadbend decoupling cycle from Malcom Levitt and incorporating the excitation sculpting sequence for water suppression. Spectra were referenced to internal 0.1 mM sodium 3-(trimethylsilyl)-2,2,3,3-tetradeuteropropionate (TSP), assumed to resonate at δ = 0.00 ppm. Two-dimensional ^1^H-^13^C heteronuclear single-quantum coherence (HSQC) spectra were recorded at 150.90 MHz for ^13^C using pre-saturation for water suppression [28]. HSQC spectra were referenced to the α-glucose doublet resonating at 5.24 ppm for ^1^H and 93.10 ppm for ^13^C.

### 2.5. Multivariate Data Analysis

The 0.50–9.50 ppm spectral region of each spectrum was automatically binned into 0.02 ppm width regions (buckets) and integrated using the AMIX 3.9.7 package (Bruker Biospin GmbH, Rheinstetten, Germany). The residual water resonance (4.40–5.60 ppm) was removed from the analyzed spectral area, and the integrated sections were normalized to the total spectrum area. To discriminate samples according to their metabolic variations, NMR profiles were studied using the Soft Independent Modeling of Class Analogy (SIMCA)14 package (Umetrics, Umeå, Sweden). Principal component analysis (PCA) and Orthogonal Projection to Latent Structures Discriminant Analysis (OPLS–DA) [29] were performed. PCA was used to reduce data dimensionality and to evaluate class homogeneity, highlighting possible clustering in an unsupervised manner. Once class homogeneity was assessed for each group, supervised OPLS-DA was applied. The quality of all PCA and OPLS–DA models was evaluated using the R^2^ and Q^2^ parameters, which represent the goodness-of-fit and the goodness-of-prediction, measuring how well the model fits the data, and how well the model predicts new data, respectively. For R^2^ and Q^2^, acceptable values must have been ≥0.5, with |R^2^ - Q^2^| < 0.2–0.3. Normality test and ANOVA test with Bonferroni correction were performed with the OriginPro 9.1 software package (Origin Lab Corporation, Northampton, MA, USA).

### 2.6. Pathway Analysis

Pathway topology and biomarker analysis were carried out using Metaboanalyst 4.0 [30]. Metabolites were selected by evaluating both variable importance in projection (VIP) values > 1 in class discrimination and correlation values |pq[corr]| > 0.7.

## 3. Results

### 3.1. Phage Xccφ1, Hydroxyapatite, and Eicosanoic Acid Modulate Xcc Biofilm

In bacterial infections, hydroxyapatite (HA) nanocrystals help in bacteriophage delivery and are reported to improve some of the bacteriophage biological properties [20]. In addition, although not bactericidal, C20:0 could be able to modify the microbial biofilm structure by altering the permeability of the cell [21,22,31,32].

It has been reported that C20:0 does not show a significant decrease in biofilm formation, especially at low concentration [33]. However, inhibition has been reported to be dose-dependent [34,35,36].

**Figure 1:**
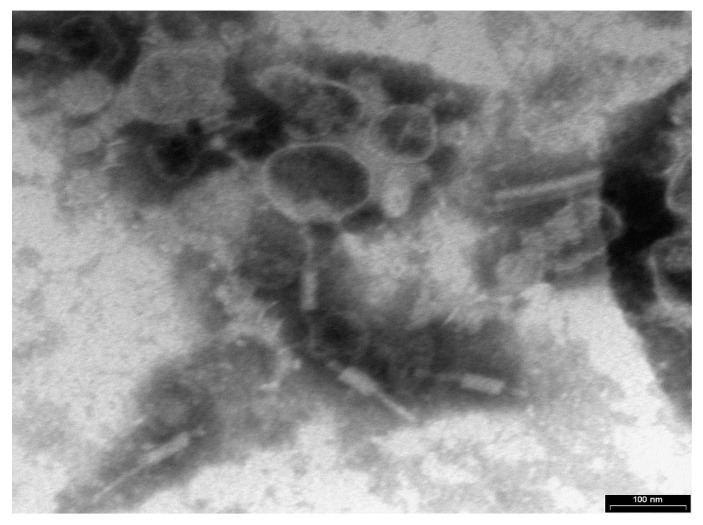
Phage Xccφ1 structure as observed by transmission electron microscopy (TEM). The scale bar represents 100 nm.

Transmission electron microscopy (TEM) examination identified Xccφ1 as a member of the Myoviridae family because of the contractile, long and relatively thick tail, with a central core separated from the head by the neck (Figure 1).

**Figure 2:**
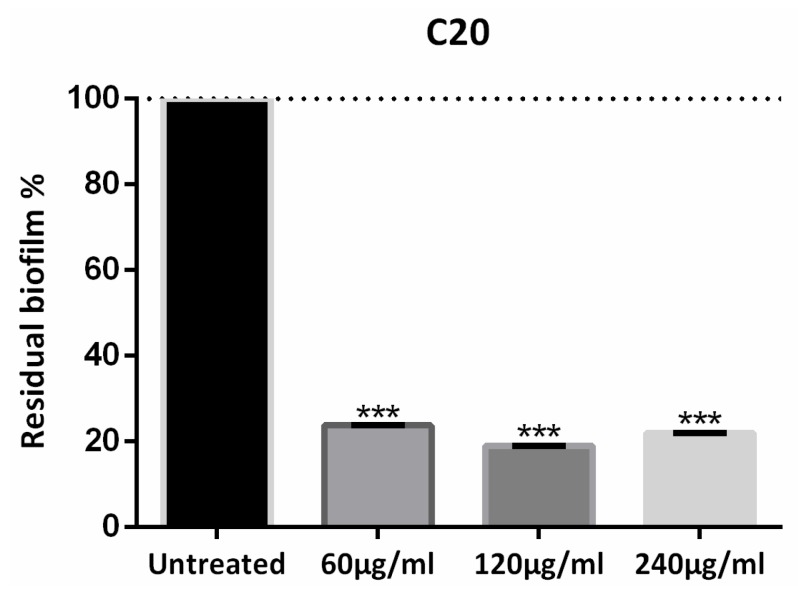
Eicosanoic acid (C20:0) activity on *Xcc* biofilm formation. Data are expressed as percent of residual biofilm. Each value indicates mean ± SD of three independent experiments. The *t* test was used to compare the absorbance of treated and untreated samples. *** *p* < 0.001.

The action of HA on the biofilm was also tested. From crystal violet measurements, we found that HA has no effect on *Xcc* biofilm (Papaianni et al., manuscript in preparation) and exerts its enhancing action [20] (building the phage and improving its lytic activity) only in the presence of the phage. The C20:0 was approximately equally active at 60 µg/mL, 120 µg/mL and 240 µg/mL—all reducing the amount of biofilm by ca. 80%. In Figure 2 the anti-biofilm effect is reported as a percentage of the residual biofilm after treatment in comparison with untreated bacteria. In the following, C20:0 was always used at the lowest active concentration of 60 µg/mL.

### 3.2. NMR Analysis: Class Discrimination

The stability of the *Xcc* biofilm upon treatment with Xccφ1, HA, C20:0, Xccφ1+C20:0 and Xccφ1+HA+C20 was analyzed by NMR-based metabolomics. We considered 10 samples for *Xcc*, Xccφ1, C20:0, and Xccφ1+HA+C20, while for HA and Xccφ1+C20:0, we analyzed six samples for each class, which amounted to 52 samples. All classes were tested by unsupervised PCA- to verify the presence of possible subgroups and/or outliers; none were detected, confirming that the classes are homogeneous. For all classes, we obtained as quality parameters 0.19 < R^2^ < 0.20 and 0.15 < Q^2^ < 0.22, with 0.61 < *p* < 0.82, which was an indication that no subgroups could be identified in the sample set. Therefore, all 52 samples (and the NMR spectra) were included in the analysis.

**Figure 3:**
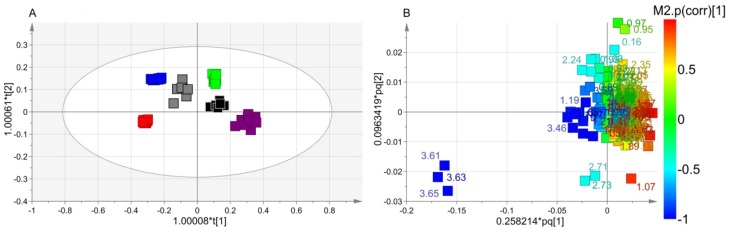
Orthogonal Projection to Latent Structure Discriminant Analysis (OPLS-DA) of *Xcc* biofilm treatment. (**A**) Scores plot showing the separation between *Xcc* (green squares), hydroxyapatite (HA) (black squares), Xccφ1+C20:0 (gray squares), C20:0 (purple squares), Xccφ1 (blue squares) and Xccφ1+HA+C20:0 (red squares). (**B**) Loadings plot reporting the nuclear magnetic resonance (NMR) variables corresponding to metabolites responsible for class separation, displaying |*p*(corr)|> 0.7.

We next applied supervised OPLS-DA to uncover metabolic differences between classes. In the scores plot of Figure 3A, the t[1] dimension identifies two groups. At negative values, we found Xccφ1, Xccφ1+C20:0, and Xccφ1+HA+C20:0 classes, while the *Xcc*, HA, and C20:0 classes were located at positive values. For such a model, we obtained good quality parameters (R^2^ = 0.68; Q^2^ = 0.75; *p* = 2.310 × 10^−20^), indicating that this was statistically significant. In particular, the scores plot data indicate that the first component highlights the effects of the phage (all treatments with phage are at negative values, while those without phage are at positive values), while the second one the effects of the C20:0 and HA on the biofilm [20].

The discriminating metabolites were identified in the associated loadings plot of Figure 3B, in which the numbers identify the NMR chemical shifts of the buckets. In particular, we considered those presenting statistical significance with variable importance in projection (VIP) values greater than 1 in class discrimination, and correlation values |*p*(corr)| greater than 0.7. With respect to the untreated biofilm, Xccφ1 induced the production of ethanol, galactomannan and glutamate and downregulated 2-aminoadipate, arginine, betaine, glycine, 3-methylhystidine, isobutyrate, isoleucine, lactate, leucine, lysine, methionine, phenylalanine, propionate, pyroglutamate, saturated fatty acids (SFAs), tyrosine and valine. With respect to *Xcc* biofilm, C20:0 presented an upregulation of arginine, dimethylamine, isobutyrate, lysine, 3-methylhystidine, pyroglutamate and tyrosine and downregulation of 2-aminoadipate, betaine, glutamate, glycine, isoleucine, leucine, methionine, phenylalanine, SFAs and valine. In comparison with *Xcc* biofilm, HA brought about an increase of dimethylamine, isobutyrate, lysine, 3-methylhystidine, and tyrosine, with a parallel reduction of betaine, glutamine, glycine, leucine, phenylalanine, and valine. Xccφ1+C20:0 amplified ethanol, galactomannan and glutamate; and reduces arginine, glycine, 3-methylhystidine, isobutyrate, lactate, leucine, lysine, methionine, phenylalanine, propionate, SFAs, tyrosine and valine. Compared to *Xcc* biofilm, the Xccφ1+HA+C20:0 class showed an increase of ethanol, dimethylamine, galactomannan and SFAs and a decrease of 2-aminoadipate, betaine, glutamate, glycine, isobutyrate, lactate, leucine, lysine, methionine, phenylalanine, propionate, pyroglutamate and valine.

Interestingly, in the phage groups (Xccφ1, Xccφ1+C20:0 and Xccφ1+HA+C20:0), the dysregulated metabolites showed the same trend, with an increasing tendency towards the Xccφ1+HA+C20:0 class.

### 3.3. Pathway Analysis

**Figure 4:**
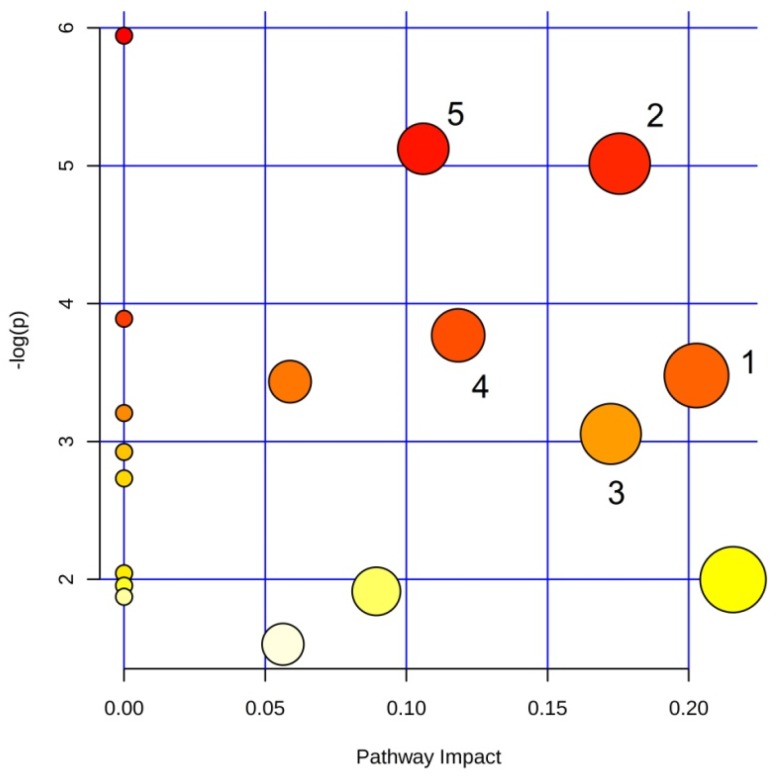
Pathway analysis based on most relevant metabolites identified by OPLS-DA. Pathways are identified as follows: 1, glycine, serine and threonine metabolism; 2, arginine biosynthesis; 3, glutamate and glutamine metabolism; 4, arginine and proline metabolism; 5, glutathione metabolism.

NMR signals with VIP >1 and |*p*(corr)| > 0.7 were used to identify the main metabolic pathways dysregulated between sample classes. Among the found pathways, the statistically significant examples were phenylalanine metabolism (labeled 1 in Figure 4; impact: 0.22); alanine, aspartate and glutamate metabolism (2; impact: 0.18); arginine and proline metabolism (3; impact: 0.17); glycine, serine and threonine metabolism (4; impact: 0.12); and glutathione metabolism (5; impact: 0.11).

## 4. Discussion

In the present study, by using NMR-based metabolomics, we investigated the metabolic changes brought about by HA, C20:0, Xccφ1, Xccφ1+C20:0, and Xccφ1+HA+C20:0 on the *Xcc* biofilm. The scores plot of Figure 3A can be interpreted as follows. The Xccφ1, Xccφ1+C20:0 and Xccφ1-HA-C20:0 classes are placed at negative coordinates of the horizontal axis (the first component t[1]), while *Xcc*, HA, and C20:0 classes are located at positive t[1]. Such a behavior derives from the presence/absence of phage, which drives the discrimination. The vertical component t[2] accounts for the separation between the Xccφ1-HA-C20:0 placed at t[2] negative coordinates in comparison with Xccφ1 and Xccφ1+C20:0 placed at t[2] positive coordinates. Such a separation can be ascribed to the presence/absence of C20:0 and HA, although a synergistic action cannot be excluded (Papaianni et al.; manuscript in preparation).

The pathway analysis identified the following dysregulated metabolic pathways involving amino acids: glycine, serine and threonine metabolism; arginine biosynthesis; glutamate and glutamine metabolism; arginine and proline metabolism; and glutathione metabolism (Figure 4). Interestingly, the amino acid metabolism is involved in the formation and maturation of the bacterial biofilm [37], and is an important energy source since it feeds the Tricarboxylic Acid Cycle (TCA).

In particular, with respect to *Xcc*, the C20:0 class, which does not include the phage, displays high levels of arginine, lysine, 3-methylhistidine, pyroglutamate and tyrosine; and low levels of glutamate, glycine, isoleucine, leucine, methionine, phenylalanine and valine. HA increases dimethylamine, isobutyrate, lysine, 3-methylhystidine, and tyrosine, with a parallel reduction of betaine, glutamine, glycine, leucine, phenylalanine, and valine. Xccφ1 shows higher glutamate, and lower arginine, glycine, 3-methylhystidine, isoleucine, leucine, lysine, methionine, phenylalanine, pyroglutamate, tyrosine and valine. Compared to *Xcc*, Xccφ1+C20:0 amplifies ethanol, galactomannan and glutamate; and reduces arginine, glycine, 3-methylhystidine, isobutyrate, lactate, leucine, lysine, methionine, phenylalanine, propionate, SFAs, tyrosine and valine. Finally, the Xccφ1+HA+C20:0 class is characterized by a decrease of glutamate, glycine, isobutyrate, lactate, leucine, lysine, methionine, phenylalanine, pyroglutamate and valine. Even though all classes affect the film, the metabolic responses involve amino acids at different levels, implying that the lytic action is exerted in different way. For example, although the C20:0 and HA do not exert a bactericidal action, they modify the microbial biofilm structure by altering the permeability of the constituent cells [21,22,31,32], and the deep dysregulation of the amino acid metabolism suggests that the biofilm cells somehow “counteract” the lytic action of both C20:0 and HA by activating/deactivating specific amino acids. On the other hand, the comparison between the effects originating from the Xccφ1, Xccφ1+C20:0 and Xccφ1+HA+C20:0 treatment indicates a similar trend in all classes, showing an increasing efficacy in the lytic action for Xccφ1+HA+C20:0. This could be due to the possible synergistic action present in Xccφ1+HA+C20:0 (Papaianni et al., manuscript in preparation), whose effects on the metabolome remains to be investigated.

The pattern of lactate is also interesting. We described here a mature (72 h old) biofilm, potentially marked by reduced levels of oxygen—a condition promoting anaerobic glycolysis and the inhibition of the TCA cycle [38]. With respect to *Xcc*, lactate was downregulated at comparable levels in both Xccφ1, Xccφ1+C20:0 and Xccφ1+HA+C20:0 classes. Lactate contributes to biofilm production [39], and added to minimal medium, it favors bacterial cell adherence to surfaces and biofilm formation [40]. Therefore, as observed, the lytic action of Xccφ1, Xccφ1+C20:0 and Xccφ1+HA+C20:0 requires reduced levels of lactate [8,41].

High levels of SFAs are observed in the classes treated with phage. Since bacteria in the biofilm state increase their membrane stability and rigidity by incorporating exogenous fatty acids into the membrane [42], the observed SFAs increase could reflect the cell lysis caused by the phage and the subsequent release of SFAs in the exogenous environment (the supernatant).

The phage classes also show high levels of galactomannan. It has been reported that xanthan and galactomannan synergistically increase the biofilm viscosity of *X. campestris* [43]. Although xanthan was not detected, galactomannan increased drastically with phage, while it remained unchanged in the C20:0 and HA classes. Galactomannan gel is unstable since loses up to 50% of its water by syneresis [44]. Thus, the absence of xanthan and the high level of galactomannan suggest that the phage reduces the viscosity of the biofilm through the production of galactomannan.

Taken together, the above results highlight the ability of the phage to dysregulate the amino acids’ metabolic pathways responsible for the formation and maturation of the bacterial biofilm, to reduce the lactate that favors biofilm production, and to upregulate the production of galactomannan that weakens the biofilm.

In conclusion, we have described here the action of Xccφ1, Xccφ1+C20:0 and Xccφ1-HA-C20:0 against *Xcc* bacterial biofilm identifying specific metabolic pathways that are dysregulated by the lytic action. Our data demonstrate that Xccφ1 alone or combined with HA and C20:0 interferes with the metabolic pathways involved in biofilm formation. The altered pathways may become the possible targets for the treatment of bacterial biofilm, as well as providing an indication of the efficacy of the treatment. The approach might be extended to the study of other biofilm-producing bacteria, such as *Escherichia coli* and *Pseudomonas aeruginosa*, in which Pf4 bacteriophage (filamentous bacteriophage) inhibits the metabolic activity of *Aspergillus fumigatus* biofilms [45], and NMR-based metabolomics could be reliably used to understand how phages act on the host metabolism.
